# Mechanical Reinforcement of ABS with Optimized Nano Titanium Nitride Content for Material Extrusion 3D Printing

**DOI:** 10.3390/nano13040669

**Published:** 2023-02-08

**Authors:** Nectarios Vidakis, Panagiotis Mangelis, Markos Petousis, Nikolaos Mountakis, Vassilis Papadakis, Amalia Moutsopoulou, Dimitris Tsikritzis

**Affiliations:** 1 Department of Mechanical Engineering, Hellenic Mediterranean University, 71410 Heraklion, Greece; 2 Department of Electronic Engineering, Hellenic Mediterranean University, 73133 Chania, Greece; 3 Institute of Molecular Biology and Biotechnology, Foundation for Research and Technology—Hellas, 71110 Heraklion, Greece; 4 Department of Electrical & Computer Engineering, Hellenic Mediterranean University, 71410 Heraklion, Greece

**Keywords:** Additive Manufacturing (AM), 3D printing, Materials Extrusion (MEX), Fused Filament Fabrication (FFF), nanocomposites, Acrylonitrile Butadiene Styrene (ABS), Titanium Nitride (TiN), mechanical properties

## Abstract

Acrylonitrile Butadiene Styrene (ABS) nanocomposites were developed using Material Extrusion (MEX) Additive Manufacturing (AM) and Fused Filament Fabrication (FFF) methods. A range of mechanical tests was conducted on the produced 3D-printed structures to investigate the effect of Titanium Nitride (TiN) nanoparticles on the mechanical response of thermoplastic polymers. Detailed morphological characterization of the produced filaments and 3D-printed specimens was carried out using Atomic Force Microscopy (AFM) and Scanning Electron Microscopy (SEM). High-magnification images revealed a direct impact of the TiN concentration on the surface characteristics of the nanocomposites, indicating a strong correlation with their mechanical performance. The chemical compositions of the raw and nanocomposite materials were thoroughly investigated by conducting Raman and Energy Dispersive Spectroscopy (EDS) measurements. Most of the mechanical properties were improved with the inclusion of TiN nanoparticles with a content of 6 wt. % to reach the optimum mechanical response overall. ABS/TiN 6 wt. % exhibits remarkable increases in flexural modulus of elasticity (42.3%) and toughness (54.0%) in comparison with pure ABS. The development of ABS/TiN nanocomposites with reinforced mechanical properties is a successful example that validates the feasibility and powerful abilities of MEX 3D printing in AM.

## 1. Introduction

Nowadays, Additive Manufacturing (AM) using 3D-printing techniques has revolutionized the industry with an increasing number of new applications, due to the ability to manufacture complex structures. It is applied in various types of applications, such as in aerospace [1], engineering [2], orthopedics [3], and electronics [4]. One of the most well-established AM methods with various applications in industry, energy, and medical engineering is Material Extrusion (MEX) [5]. Several studies have shown that appropriate MEX parameters and conditions are critical for the 3D-printing process because they significantly affect the properties and mechanical performance of the produced specimens [6,7,8]. From the point of view of materials science and chemical engineering, novel materials with appropriate thermal, mechanical, and physical properties, specifically designed for 3D-printing applications, are required to enhance the mechanical performance and functionality of 3D-printed structures. Thermoplastic materials, such as Acrylonitrile Butadiene Styrene (ABS), are widely used for MEX 3D-printing processes, specifically for fused filament deposition processes, because of their superior mechanical and physical properties [9,10,11].

ABS is a triblock copolymer from a group of styrene terpolymers and exhibits good mechanical properties such as strength and toughness combined with high impact and resistance to heat and scratching. It is also a low-cost and easy-processing material for 3D-printing applications [12]. In addition, ABS is characterized as a sustainable and fully recyclable material because it exhibits excellent mechanical response over multiple recycling processes [13]. These characteristics make ABS an advantageous material that has been used in different sectors such as the automotive industry, electronics, construction, and machines [14,15,16,17]. The mechanical properties of MEX 3D printing have been thoroughly investigated, with studies focusing on its response to different strain rates [18,19], tensile tests [20,21,22], compression [23], and creep [24], among others. The effect of 3D-printing parameters on the performance of build parts has been studied, with experimental results being optimized using modeling tools [25].

Many studies have shown that the development of polymer composites using MEX 3D-printing methods and introducing additives with exceptional properties (electrical, mechanical, thermal, optical, etc.) in polymeric matrices can reinforce the mechanical and physical properties of composites compared to those of pure polymers or even provide new functionalities because the advantageous properties of additives are incorporated in the 3D-printed structures [26,27,28,29,30,31,32,33,34]. Such an approach can further expand the fields of application of the process in the engineering, biomedical, and electronics fields, among others [35]. The introduction of catalytic ΤiO_2_ nanoparticles in ABS thermoplastic triggered the chemical reactivity of developed 3D-printed filaments while simultaneously the breaking point stress was raised to a higher level [36]. ΤiO_2_ nanoparticles have been used as an additive in the ABS matrix in MEX 3D printing for the enhancement of the mechanical properties in other research works as well [37,38].

Recently, Huang et al. developed ABS nanocomposites introducing a multifunctional graphene-based filler, achieving smoke suppression and flame retardation while in parallel the tensile strength was increased up to 31% and the elastic modulus up to 73% [14]. Several studies have reported the addition of carbon-based additives to the ABS matrix to exploit the remarkable electrical transport and storage properties of conductive nanofillers, aiming to develop conductive polymer nanocomposites using different AM processes [15,39,40]. Superior reinforcements in electrical conductivity and mechanical properties were exhibited by ABS/MWCNT nanocomposites developed by fused filament deposition modeling [41,42]. By dispersing carbon black in ABS through melt compounding, improved thermal stability was achieved [43]. Metal/Polymer composites have also been developed for MEX 3D printing, using ABS as the polymeric material in the composites [44].

Moreover, melt blending of appropriate materials with ABS, degradation studies, and thermomechanical investigations have been reported [45,46,47]. The thermal and mechanical behavior of ABS nanocomposites has also been enhanced by introducing polymer matrix metal fillers such as iron and copper [48,49]. Superhydrophobic ABS nanocomposites were developed by introducing silica nanoparticles via solution processing and spray coating, and as a result, a notable improvement in wear abrasion resistance was achieved [50]. In a previous study by this team, 3D-printed ABS-based composites were obtained by adding ZnO particles at the nano- and micro-scales and using the fused filament fabrication (FFF) process. Mechanical tests have shown effective enhancements in the tensile and flexural strengths of both nano- and micro-composite materials [51]. ABS has also been investigated in Hybrid Additive Manufacturing, combining MEX 3D printing and friction stir welding for manufacturing large parts [52], or MEX 3D printing and laser cutting for the improvement of the quality characteristics of the parts built with the process [53].

Several reports have shown that nitride nanoparticles are exceptional additives for the development of polymer nanocomposites, providing effective reinforcement for thermomechanical performance [54,55]. Titanium nitride is a hard ceramic material that has attracted particular interest because of its exceptional wear, erosion, and corrosion resistance [56]. Titanium Nitride (TiN)-based coatings have been applied to metallic parts of components, machining, and cutting tools to improve their wear resistance and lifetime [57,58]. Polycrystalline TiN thin films have also been used as diffusion barrier layers in IC technology [59], and TiN nanosheets have been coated on zinc electrodes to improve the cycle performance of high-energy rechargeable batteries [60]. Moreover, TiN has been investigated for medical applications as an abrasion- and corrosion-resistant coating on dental implants [61,62], while TiN-coated orthopedic implants have demonstrated effective biocompatibility and tribological properties [63]. Studies have also shown that TiN exhibits attractive optical properties and plasmonic performances [64,65,66,67]. There are very few reports on the use of TiN as an additive for the development of nanocomposites using MEX 3D printing. Recently, PC/TiN nanocomposites were successfully developed by this team using the FFF 3D-printing method. Our study showed an effective enhancement of the mechanical properties of the produced specimens with the inclusion of small amounts of TiN nanoparticles.

Ceramic additives such as TiN are very popular enhancement agents, as shown in the literature review above, and MEX 3D printing has not been applied. Therefore, this study investigated the effect of TiN as an enhancement agent for the ABS polymer in MEX 3D printing. To date, no similar research has been conducted. The proposed idea is to use the TiN filler in nanoparticles form to enhance the mechanical properties of the second most popular polymer in MEX 3D printing, which is ABS. Such polymers with enhanced mechanical properties are highly desirable in MEX 3D printing, as they further extend the application fields of the process, exploiting its advantages, such as the ability to manufacture parts without limitation in their geometry. TiN is a common ceramic for enhancing the performance of materials, especially in coatings and elsewhere, as shown in the literature review. Still, it has never been used before as an additive in nanocomposites to act as an enhancing agent for the ABS polymer in 3D printing. Thus, such nanocomposites are developed with the process followed and investigated for their effect as enhancing agents for the first time in the literature.

In an effort to expand the utilization of TiN as a nanofiller in other polymeric matrices and to investigate its impact on the mechanical response of new nanocomposite materials, ABS/TiN nanocomposites were fabricated for the first time using MEX (fused filament fabrication—FFF) 3D printing. The effect of TiN nanoparticles on the mechanical properties of the produced filaments and 3D-printed specimens has been studied extensively. The mechanical response of the ABS/TiN nanocomposites was comprehensively evaluated and optimized as a function of the additive concentration. The thermal stability of the developed nanocomposites was studied by Thermogravimetric Analysis (TGA), and Raman and Energy Dispersive Spectroscopy (EDS) measurements were carried out to investigate and identify the chemical and elemental composition. Mechanical tests were performed according to the American Society for Testing and Materials (ASTM) standards. Moreover, morphological studies were performed using Atomic Force Microscopy (AFM) to investigate the surface morphology of the produced filaments, while Scanning Electron Microscopy (SEM) was used to study the morphological characteristics of the final 3D-printed specimens and to evaluate the overall MEX printing process. An ABS/TiN nanocomposite with six weight-to-weight percentages (wt. %) additive content exhibits the highest improvements in most mechanical properties with remarkable reinforcements of tensile and flexural strengths, validating, finally, a new success story for the development of ABS nanocomposites by the powerful MEX 3D printing. Such materials with enhanced mechanical performance are highly sought after in MEX 3D printing, and they further expand the field of application of this process.

## 2. Materials and Method

**Figure 1:**
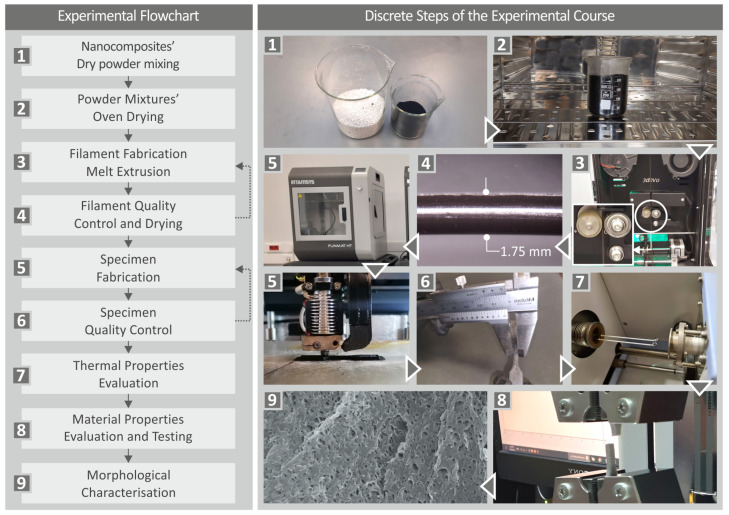
Workflow of experimental procedure: Flowchart of the experimental process and screenshots of the corresponding experimental steps followed.

Figure 1 presents the steps of the experimental procedure followed for the fabrication of the investigated specimens, as well as their thermomechanical and morphological characterization.

### 2.1. Materials

The nanocomposite specimens were fabricated using the ABS polymer as the matrix material in the form of powder (Terluran Hi-10, INEOS Styrolution, Frankfurt, Germany) with an ultimate tensile strength of 38 MPa and density of 1080 kg/m^3^. Titanium nitride (TiN) in nanopowder form with a size of 20 nm was used as an additive purchased from Nanographi (Ankara, Turkey, purity 99.2%, melting point at 2950 °C and density of 0.08 g/cm^3^).

### 2.2. Nanocomposites Preparation

**Figure 2:**
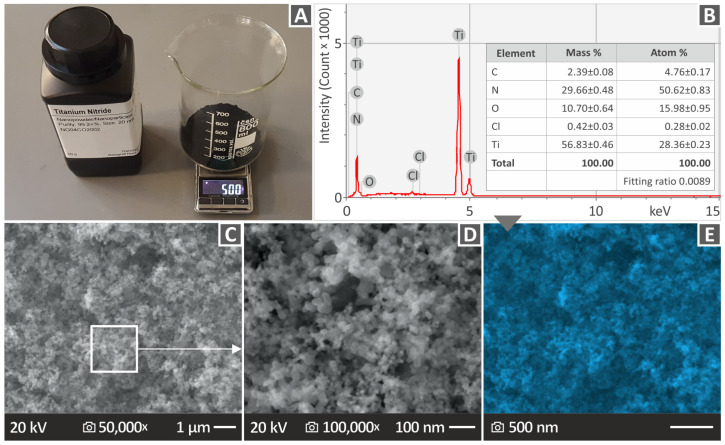
TiN nanopowder examination (**A**) TiN powder weight for correct wt. % concentration in the nanocomposites, (**B**) EDS graph, (**C**) 50,000× SEM image, (**D**) 100,000× SEM image, (**E**) EDS mapping for the Ti element.

Scanning Electron Microscopy (SEM) and Energy Dispersive Spectroscopy (EDS) analyses of the purchased nanopowder were conducted to evaluate the characteristics of the material before nanocomposite preparation. SEM images (Figure 2C,D) verified the size and shape of the TiN nanoparticles. As can be observed in Figure 2B, the peaks with the highest intensities are attributed to the elements of Ti and N. However, oxygen was also identified with a mass percentage of 10.7%, while chlorine and carbon traces were detected in quite slight amounts. The presence of oxygen may have originated from moisture. Figure 2E shows the EDS mapping for the titanium element in the TiN nanoparticles. As it is shown, the dispersion found with the EDS mapping process, of the titanium element in the nanoparticles is uniform, without voids, and areas with differences in the titanium concentration and agglomerations. Titanium was located throughout the observation area. Regarding the EDS graph, the elements found in the nanoparticles were the expected ones from the composition of the TiN powder, according to the manufacturer. As expected, Ti peaks are higher indicating a higher concentration of the element in the observation region.

The pure materials were dried for 14 h in a dryer at 60 °C to remove moisture. Five different mixtures of raw materials were prepared by weight-to-weight (wt. %) TiN ratios of 1.0, 2.0, 4.0, 6.0, and 8.0 wt. %. This was the concentration by weight of the TiN nanoparticles in the nanocomposites, the remaining amount was the ABS polymer in each nano-compound (99.0, 98.0, 96.0, 94.0, and 92.0 wt. % respectively) and no other additives were used in the nano compounds. For the mixing of TiN nanoparticles with the ABS matrix, a high-power blender was used at 4000 rpm for 30 min at room temperature (23 °C) to obtain an initial dispersion of TiN nanoparticles in the ABS matrix. A second drying of the prepared mixtures was then performed. A single-screw Noztek (Shoreham-by-Sea, UK) extruder was used for filament fabrication, and a 3devo shredder (3devo B.V., Utrecht, The Netherlands) was used for filament fabrication. The fabricated pellets were processed using a 3devo Composer (3devo B.V., Utrecht, The Netherlands). This extruder is equipped with a special screw design for material mixtures to produce appropriate filaments for 3D MEX printing with a diameter of 1.75 mm. The three heating zones 1–3 were operating at 200 °C, whereas the temperature of the fourth heating zone was set at 240 °C. Screw rotation was set at 4.8 rpm.

The implementation of two extrusion procedures targeting to enhance the dispersion of TiN nanoparticles in the polymeric matrix, while it must be mentioned here that no compatibilizers or other fillers were added during the preparation process of nanocomposites to effectively investigate the direct impact of TiN nanoparticles in ABS matrix. The same extrusion process was followed for filament production of pure ABS without TiN content. This was necessary for a comparative study of the mechanical responses of the nanocomposites.

### 2.3. Fabrication of 3D-Printed Specimens

**Figure 3:**
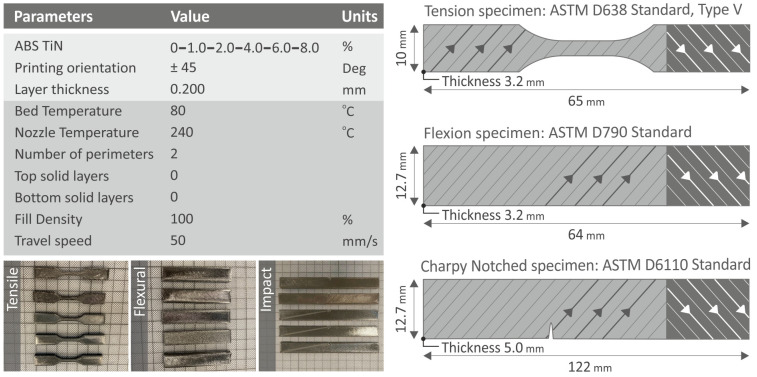
3D-printing parameters for the manufacturing of specimens. The specified dimensions of fabricated specimens for each mechanical test are presented on the right side, along with the respective international standard the specimens are confronting, and it was followed in the work.

A Funmat HT 3D printer (Intamsys, Shanghai, China) was used to fabricate specimens from the filaments of pure polymer and ABS/TiN nanocomposites produced by the previous two extrusion processes. G-codes were extracted using the Intamsuite software platform (Intamsys, Shanghai, China). Figure 3 presents the 3D-printing setup parameters that were determined experimentally before the 3D-printing process, as well as the produced specimens with dimensions specified according to ASTM standards for each test: ASTM D638-02a, ASTM D790-10, and ASTM D6110-02 for tensile, flexural, and Charpy notched impact tests, respectively. The infill pattern used to manufacture the samples is also shown in Figure 3. One infill pattern was used, that is the rectilinear one. The direction of the raster is changing between successive layers between two distinct values, i.e., +45 deg and −45 deg. Thus, in every other layer in the 3D-printing structure, the raster direction is the same.

### 2.4. Thermogravimetric Analysis and Raman Spectroscopy Measurements

The thermal stability of the nanocomposites was investigated by carrying out TGA measurements. A Perking Elmer Diamond (PerkinElmer, Inc., Waltham, MA, USA) was used for the TGA measurements, ranging from room temperature to 550 °C, with a 10 °C/min step. Measurements were performed under an N_2_ atmosphere.

Raman spectroscopy measurements were carried out using a HORIBA LabRAM HR spectrometer (HORIBA Scientific, Kyoto, Japan) to study the chemical bonds of the pure ABS and the developed nanocomposites. The microscope features a solid-state laser at 532 nm with a maximum output power of 90 W. For the measurements, a 50× magnification objective lens was used with a numerical aperture of 0.5, and the working distance was regulated at 10.6 mm. The dimensions of the laser spot on the samples were ca. 1.7 μm laterally and ca. 2 μm axially. A neutral density filter controlled the laser power to 2 mW, enabling 5% of laser light to pass through. Data were collected using two acquisitions of 10 s per point and five accumulations per measurement in a spectral range between 300 and 3100 cm^−1^ with a resolution of 2 cm^−1^. The Raman spectra were processed using LabSpec 6 software (HORIBA Scientific, Kyoto, Japan). The background was subtracted using a polynomial function and a Unit Vector was used to normalize the data of the investigated samples.

### 2.5. Evaluation of Produced Filaments

Before the start of the 3D-printing process and the fabrication of specimens, all produced filaments underwent diameter and tensile measurements, as well as surface characterization. The diameter of all filaments was measured in real-time during the extrusion process using a closed-loop measuring system, while after 3D printing, a cross-check of the diameter results was carried out using an electronic caliper. Tensile strength measurements were performed using an Imada MX2 instrument (Imada Inc., Northbrook, IL, USA). Standard grips were used to fix the filaments in the instrument, while all tests were carried out at the same speed for the dogbone specimen tests at 10 mm/min. Five filament samples were tested for each nanocomposite. The side surface morphology was also studied by conducting AFM measurements to evaluate the quality of the filaments produced, as well as the MEX process. Atomic force microscopy (AFM) measurements were performed in the air using an XE7 AFM instrument (Park Systems, Seoul, Republic of Korea). Images were acquired using a PPP-NCHR cantilever (Nanosensors, USA) with a tip with a nominal diameter of 10 nm. Image acquisition was performed at a drive frequency of approximately 300 kHz. Intermittent contact mode was used to record the image, using a scan rate of 0.5 Hz. The working set point was above 70% of the free oscillation amplitude.

### 2.6. Mechanical Characterization

A range of mechanical tests was carried out according to ASTM standards to study the mechanical response of 3D-printed specimens and the effect of TiN nano-additives on the mechanical properties of thermoplastic polymers. Table 1 shows analytically all tests conducted under ambient conditions (23 °C and 55% humidity), as well as the parameters set for each test. Five specimens for each investigated ABS/TiN nanocomposite were fabricated and tested.

### 2.7. Morphological Characterization of 3D-Printed Specimens

The fracture and side surface morphologies of the produced specimens were investigated thoroughly by conducting SEM with a JEOL JSM 6362LV (Jeol Ltd. Tokyo, Japan) operating at 20 kV under a high vacuum. SEM images were collected for the gold-sputtered specimens at different magnifications. EDS analysis was also carried out in all uncoated nanocomposites, as well as in the pure polymer, to confirm the elemental composition of the investigated materials.

## 3. Results

### 3.1. Thermogravimetric Analysis and Raman Spectroscopy Measurements

**Figure 4:**
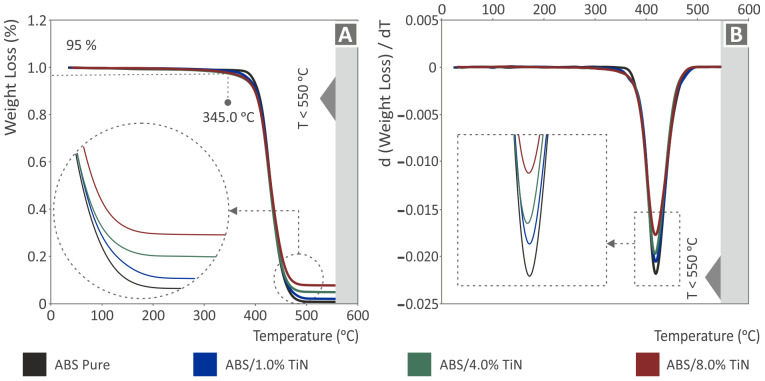
TGA graphs for pure ABS and ABS/TiN nanocomposites depicting (**A**) the weight loss and (**B**) its derivative for temperatures up to 550 °C.

TGA diagrams of the investigated nanocomposites as well as of the pure ABS are shown in Figure 4, where the weight loss is depicted as a function of temperature. As can be seen, the introduction of TiN nanoparticles in the ABS matrix does not affect the thermal stability of the matrix material. As a result, the weight loss of all nanocomposites starts at 345.0 °C, which is quite close to that of the pure ABS polymer. This temperature was much higher than the temperature settings applied during the extrusion processes (filament production and MEX 3D printing). It must be noted here that the decrease in weight for the pure ABS is more abrupt than that of the nanocomposites. This can also be observed in Figure 4B, where the maximum weight loss rate of pure ABS reached the highest value (*ca. -*0.0225), while this rate for the nanocomposites is reduced gradually with an increase in the TiN content. Therefore, ABS/TiN 8.0 wt. % exhibits the lowest absolute value close to 0.01. However, the temperature region where the maximum weight loss rate occurs for both the pure ABS and nanocomposite materials does not change, which is in agreement with previous observations.

**Figure 5:**
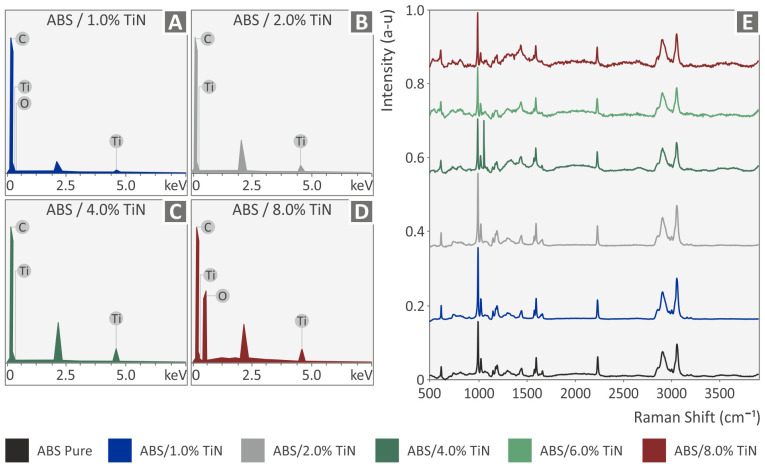
EDS analysis for ABS/TiN nanocomposites with (**A**) 1.0 wt. % TiN, (**B**) 2.0 wt. % TiN, (**C**) 4.0 wt. % TiN, and (**D**) 8.0 wt. % TiN. (**E**) Raman spectroscopy data for pure ABS and all ABS/TiN nanocomposites.

Figure 5A,D present the elemental analysis of the four investigated nanocomposite materials provided by the EDS measurements. As can be observed, titanium is identified in all samples with higher percentages as TiN content increases in the samples. The unusual peak is close to 0.6 keV, which is observed for the 8.0 wt. % TiN sample, and is attributed to oxygen. This was probably due to the presence of moisture. Raman spectroscopy data are shown in Figure 5E for pure ABS and all ABS/TiN nanocomposites. As can be observed, there are no differences between the Raman spectroscopic data of all the developed nanocomposites and those of the pure ABS polymer. All peaks presented up to 3000 cm^−1^ are attributed to the modes of the ABS matrix. It should be noted that the peak appearing at 1062 cm^−1^ is due to cosmic ray noise. It was decided not to remove this peak, since the removal algorithm was affecting also the intensity of the peaks at 1001 cm^−1^.

### 3.2. Filament Evaluation

**Figure 6:**
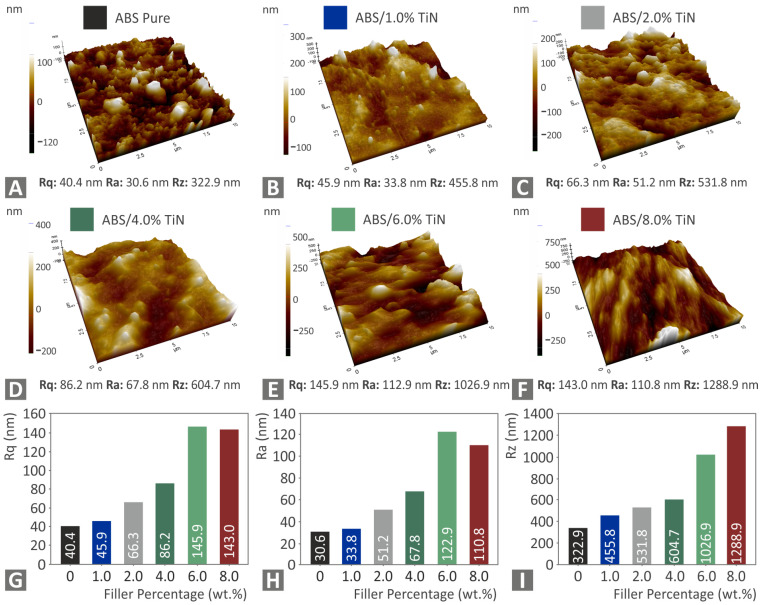
AFM images of the side surface of the tested filaments for (**A**) pure ABS (**B**) ABS/1 wt. % TiN (**C**) ABS/2 wt. % TiN, (**D**) ABS/3 wt. % TiN, (**E**) ABS/6 wt. % TiN, and (**F**) ABS/8 wt. % TiN, and graphs for the surface roughness parameters vs. the TiN concentration in the nano-compounds (**G**) Rq, (**H**) Ra, (**I**) Rz.

The side surface morphologies of all fabricated filaments were studied using AFM. As can be observed in Figure 6, all the filaments of the nanocomposites exhibit higher surface roughness values than those of the pure ABS with high TiN content samples, showing an increase of more than one order of magnitude. It is obvious that the inclusion of the TiN additive in the ABS matrix has a strong impact on the morphology of the produced filaments because the surface roughness presents an almost linear gradual increase with an increase in the TiN content up to 6 wt. %. For a high TiN content above 6 wt. %, the morphology of the tested filaments is not affected greatly by the nanopowder inclusion, as the surface roughness values seem to reach a threshold. Figure 6G–I shows graphs for the three surface roughness, i.e., Rq, Ra, Rz, as functions of the TiN concentration in the nano-compounds. As is shown, all three surface roughness metrics increase with the increase of the TiN concentration in the nanocomposites. This increase in the surface roughness on the side surface of the filament shows a reduction in the filament quality, which also may negatively affect the processability of the filament during the 3D-printing process.

**Figure 7:**
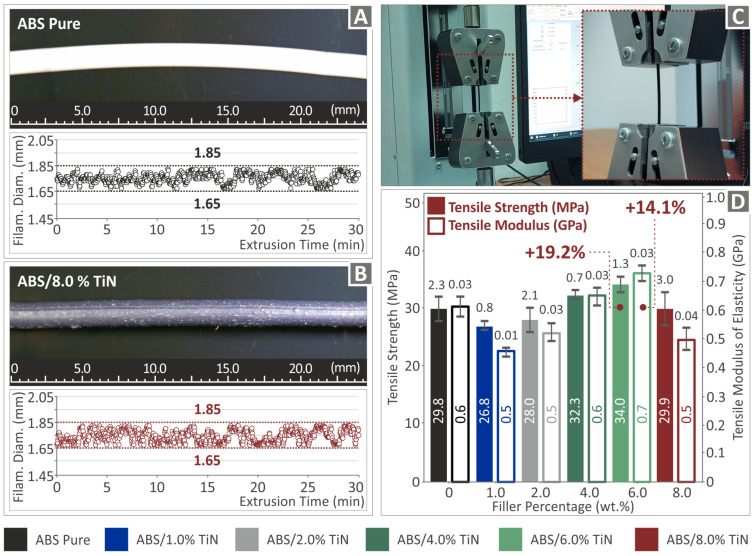
Real-time image and diameter measurements of the filament segment produced by the extrusion process for (**A**) pure ABS and (**B**) ABS/TiN 8.0 wt. % nanocomposite (**C**) experimental setup for the tensile test measurements and (**D**) results for the tensile strength and the tensile modulus of elasticity of the produced filaments.

The tested filaments were extruded using a 3devo composer (3devo B.V., Utrecht, The Netherlands) extruder equipped with a closed-loop filament diameter system, as previously mentioned. The integrated system can measure the diameter of the produced filaments in real time and automatically regulate the settings of the extrusion process within an acceptable limit, thereby enabling the production of a homogeneous filament with sufficient accuracy in diameter. Figure 7A,B show two randomly selected segments of the produced filaments (images taken with an optical stereoscope, OZR5, KERN & SOHN GmbH, Albstadt, Germany), as well as real-time diameter measurements for pure ABS and ABS/TiN 8 wt. % nanocomposites, respectively. As can be observed, the diameter values of both filaments produced present a quite low deviation (200 µm), which is acceptable for MEX 3D printing. It should be noted that during the extrusion process for all the produced filaments, the real-time diameter measurements did not exceed this representative deviation, validating the accuracy of the experimental process and the correct determination of the setting parameters. In the optical stereoscopic images (Figure 7A,B), a smooth surface was observed in the filaments without defects. In the images of the 8 wt. % TiN concentration nanocomposite (Figure 7B), the surface seems to be rougher, which was verified in the AFM measurements.

Tensile tests of the filaments produced were conducted using the setup illustrated in Figure 7C. The results of the tensile tests are shown in Figure 7D. Initially, at low additive concentrations (up to 2 wt. %, a slight decrease is observed in the tensile strength of the nanocomposite filaments compared to that of the pure polymer. However, for TiN concentrations greater than 2 wt. %, a clear enhancement of the tensile strength is achieved, reaching a threshold of 6 wt. % TiN content with a maximum increase of 19.2%. The stiffness of the filaments produced was also affected by the addition of TiN nanoparticles. Again, the maximum value is exhibited by the ABS/TiN 6 wt. % nanocomposite, which is 14.1% higher than that of the pure ABS material.

### 3.3. Mechanical Tests of the 3D-Printed Specimens

**Figure 8:**
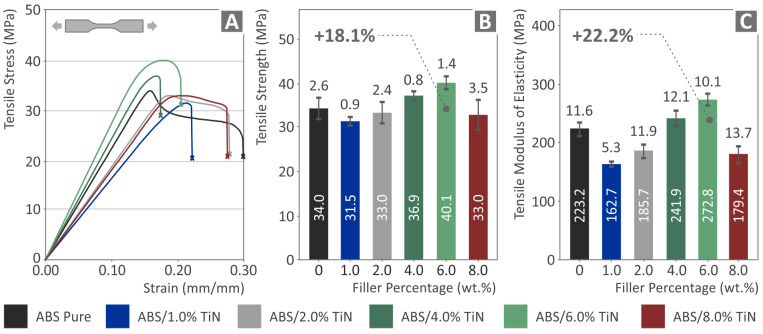
Tensile tests of the six 3D-printed specimens: (**A**) tensile stress vs. strain graph of one randomly selected specimen of the five tested in each nano compound, average values, and deviation of the (**B**) tensile strength results, and (**C**) tensile modulus of elasticity results.

After the evaluation of the filaments produced, 3D printing of the specimens was performed to study their mechanical performance. Figure 8 shows the results of the tensile tests. As can be observed, the low additive concentration samples present a decrease in the tensile strength and stiffness compared to the pure ABS polymer, whereas above 2 wt. % TiN, an enhancement of both the tensile properties is observed which follows a similar trend to that of the tensile tests of the produced filaments. A nanocomposite with 6 wt. % TiN exhibits the highest increase in both the tensile strength and stiffness, with percentages of 18.1% and 22.2%, respectively. Above this point, the decrease follows values lower than those of the pure ABS polymer. This indicates that a threshold of 6 wt. % TiN and the aforementioned mechanical properties have reached their optimum values as a function of TiN wt. %.

**Figure 9:**
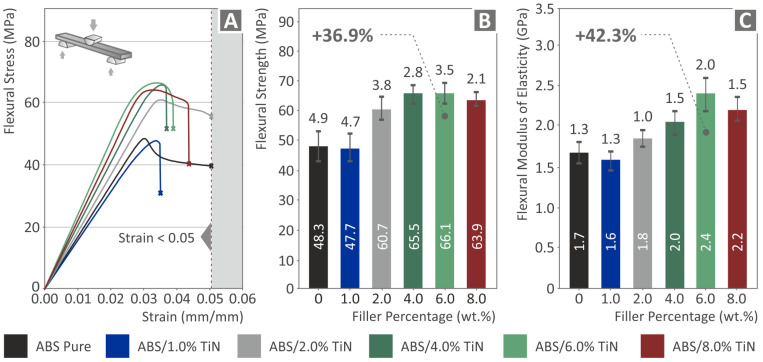
Flexural tests of the six 3D-printed specimens: (**A**) flexural stress vs. strain graph of one randomly selected specimen of the five tested in each nano compound (experiment termination: 5% strain, as per the ASTM D790 standard), average values and deviation of the (**B**) flexural strength results and (**C**) flexural modulus of elasticity results.

The flexural test results are shown in Figure 9. The addition of TiN nanoparticles to the ABS matrix strongly affected the reinforcement of the flexural properties. Above 1 wt. %, all nanocomposites demonstrate higher values than that of pure polymer, and a gradual increase in the flexural strength is observed as a function of the TiN concentration, reaching a threshold at 6 wt. %. The ABS/TiN 6 wt. % nanocomposite exhibits the maximum flexural strength value of 66.1 MPa, which is 36.9% higher than that of pure ABS. A similar trend was observed for the flexural modulus of elasticity, where all nanocomposites with TiN contents above 1 wt. % present improved mechanical response compared to the unloaded polymeric matrix. In this case, the nanocomposite with 6 wt. % exhibits the optimum value of 2.4 GPa, achieving a notable increase of 42.3% compared to that of pure ABS.

**Figure 10:**
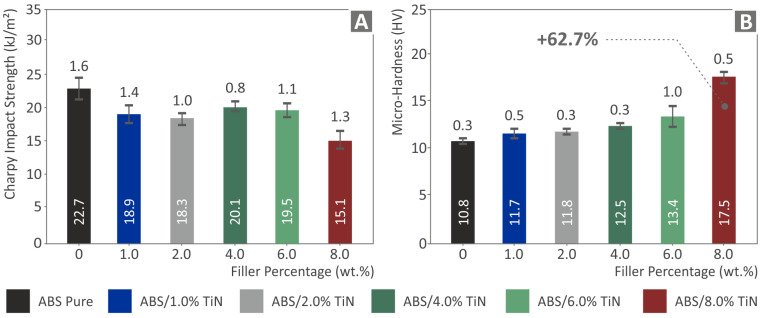
Results (average values and deviation) from (**A**) Impact strength and (**B**) Vickers microhardness tests of the six 3D-printed specimens.

Figure 10 shows the results of the impact strength and Vickers microhardness tests. As can be observed, the impact strength of the nanocomposites presents a completely different trend as a function of TiN content in comparison with the previous mechanical properties. Here, the addition of the TiN filler in the polymeric matrix had a negative effect on the mechanical response of the material, reducing the impact strength of all tested nanocomposites compared with that of the pure ABS polymer. However, the microhardness results clearly improved for all tested nanocomposites, demonstrating values higher than that of the unloaded polymeric matrix. A gradual increase in microhardness was observed throughout the ABS/TiN wt. % nanocomposites with the increase of the additive concentration. Nanocomposites with 8 wt. % TiN exhibit a remarkable reinforcement of their microhardness, reaching the value of 17.5 HV, which is 62.7% higher than that of the pure ABS polymer.

**Figure 11:**
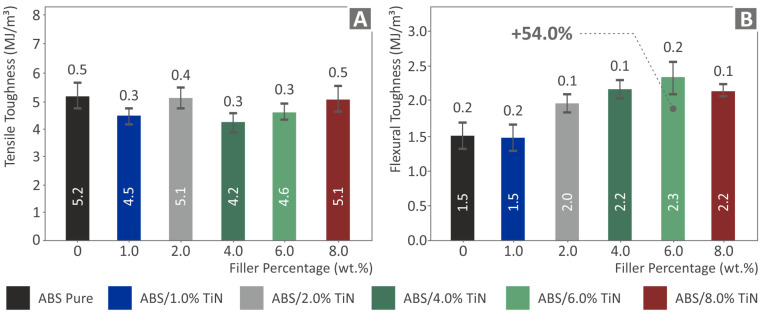
Average values and deviation of the (**A**) Tensile toughness and (**B**) flexural toughness results for the six 3D-printed specimens.

The integrals of the stress versus strain graphs were used to extract the values of the tensile and flexural toughness, which indicate the energy absorbed by the materials during the tests. As shown in Figure 11, the tensile toughness values present a slight reduction for all the developed nanocomposites compared with the pure ABS polymeric matrix. However, it should be noted that the nanocomposites with 2 wt. % and 8 wt. % TiN loading demonstrate values quite close to that of the ABS polymer. In contrast, the flexural toughness of the nanocomposites presents an important improvement, as the TiN concentration increases in the ABS matrix material. Above 1 wt. % TiN, a gradual increase is observed, reaching a threshold at 6 wt. % TiN. As a result, the ABS/TiN 6 wt. % nanocomposite exhibits a remarkable increase, with a percentage of 54% compared to the pure ABS polymer. The mechanical test results are summarized in Table 2.

### 3.4. Morphological Characterization of the 3D-Printed Specimens

**Figure 12:**
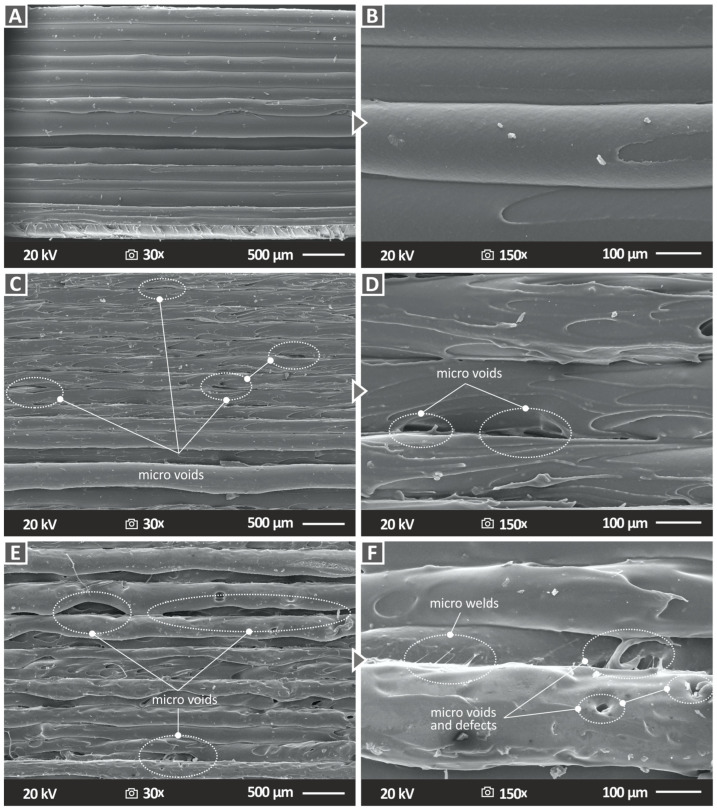
Side surface SEM images for (**A**) pure ABS at 30× magnification, (**B**) pure ABS at 150× magnification (**C**) ABS/TIN 4 wt. % at 30× magnification (**D**) ABS/TIN 4 wt. % at 150× magnification (**E**) ABS/TIN 8 wt. % at 30× magnification (**F**) ABS/TIN 8 wt. % at 150× magnification.

Subsequently, morphological characterization was performed using SEM to study the sides and fracture surfaces of the 3D-printed specimens. Figure 12 presents the side surface images of the pure ABS polymer and two representative nanocomposite samples, ABS/TiN 4 wt. % and ABS/TiN 8 wt. %, at two different magnification levels. As can be observed for the pure ABS specimen (Figure 12A,B), the side surface demonstrates perfect layer interfusion without defects or voids, validating the high quality of the 3D-printing procedure and the compatibility of the ABS polymer with the applied setting parameters. In contrast, for the nanocomposite specimens, the SEM side surface images revealed some microvoids. More interestingly, when comparing Figure 12C,E, it is revealed that the size of the voids seems to be correlated with the concentration of TiN nanoparticles in the polymeric matrix because the voids are larger in the case of the nanocomposite with 8 wt. % TiN. Moreover, a 150× magnification image of the ABS/TiN 8 wt. % (Figure 12F), apart from voids, micro welds, and defects, are detected in the side surface of the specimen, which may negatively affect the mechanical behavior of the specific sample [68]. For ABS/TIN 4 wt. %, in contrast, no micro welds or defects are detected on the side surface of the specimen.

**Figure 13:**
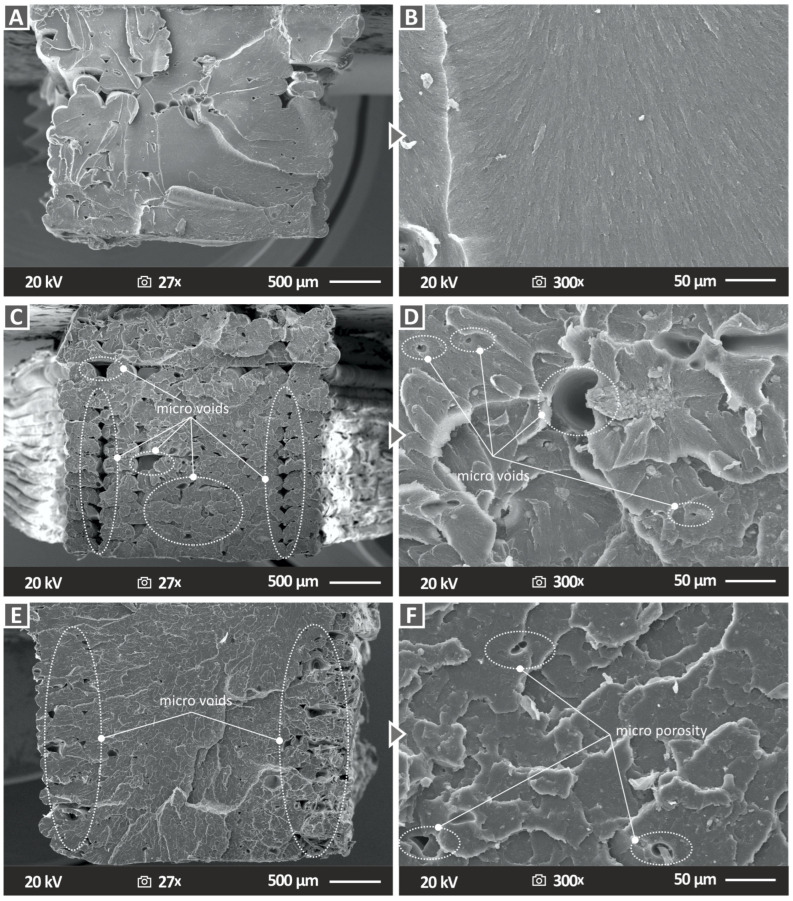
Fracture surface SEM images for (**A**) pure ABS at 27× magnification, (**B**) pure ABS at 300× magnification (**C**) ABS/TIN 4 wt. % at 27× magnification (**D**) ABS/TIN 4 wt. % at 300× magnification (**E**) ABS/TIN 8 wt. % at 27× magnification (**F**) ABS/TIN 8 wt. % at 300× magnification.

Figure 13 illustrates the corresponding fracture surface images of the investigated samples at two different magnification levels. For the pure ABS polymer, no defects or microvoids were observed on the fractured surface. In contrast, microvoids were detected in the nanocomposites, which were located mostly in regions close to the edges of the two specimens. The nanovoids are present in the specimen due to the layer-by-layer 3D-printing structure of the MEX 3D-printed parts and they are expected in the 3D-printing structure even at the 100% infill ratio [69]. It should be noted that these voids are not in the filament. If these voids were in the filament, the overall efficiency would be affected. During the inspection of the produced filament (Figure 7), before the MEX 3D-printing process, no voids or defects were located in the filament. This indicates that the extrusion process was successfully completed and the parameters used were appropriate for the specific nanocomposites. Thus, the overall efficiency was not affected by the quality of the filament in the study.

In addition, the SEM images of the ABS/TIN 8 wt. % nanocomposite at 300× magnification (Figure 13F) show some micro-porosity in the fracture surface of the specimen [69]. This verifies the claim of moisture absorption in the sample.

**Figure 14:**
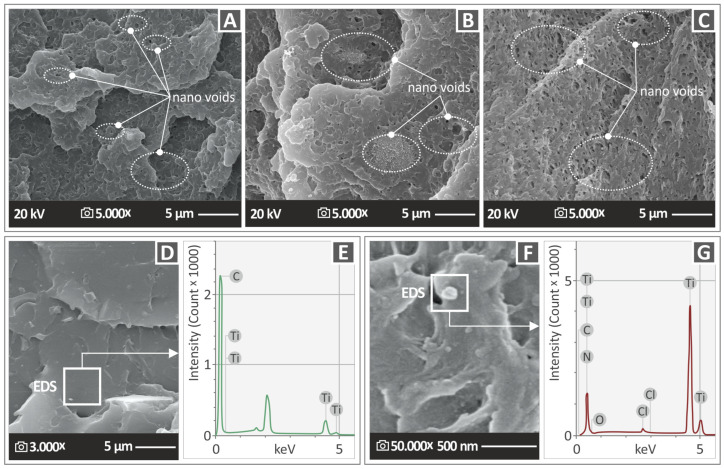
Fracture surface SEM images of specimens at 5000× magnification for (**A**) ABS/TiN 1 wt. % (**B**) ABS/TiN 4 wt. %, (**C**) ABS/TiN 6 wt. %. (**D**) ABS/TiN 6 wt. % at 3000× magnification and (**E**) EDS analysis of ABS/TiN 6 wt. % obtained on a random region of the side surface image. (**F**) ABS/TiN 8 wt. % at 50,000× magnification and (**G**) EDS analysis for ABS/TiN 8 wt. % obtained on a TiN nanoparticle-rich region.

In order to enlighten in-depth the fracture surface of 3D-printed specimens and to extract conclusions about the impact of TiN nanoparticle inclusion on the morphology of the ABS matrix, high-magnification (5000×) SEM images were captured for the nanocomposite specimens with 1 wt. %, 4 wt. % and 6 wt. % TiN content (Figure 14A–C). The images reveal nanovoids in the three specimens [70]. The illuminating feature here is that the quantity and size of the nanovoids have a clear correlation with the concentration of the TiN nanoparticles. The 1 wt. % TiN sample presents less and smaller in size nanovoids compared to those of the 4 wt. % TiN, while the highest-content sample shows a dense distribution of voids on the surface with also a larger size distribution.

Nanovoids probably affect the nanocomposite porosity, which seems to be enhanced with an increase in the TiN concentration in the polymeric matrix. Figure 14D shows a side surface image of the nanocomposite specimen with 6 wt. % TiN. As can be observed, no voids or defects are detected on the surface. In addition, there were no indications of TiN nanoparticle agglomeration. Figure 14E shows the EDS elemental analysis carried out in the region of the previous image. EDS confirmed that the peak with the highest intensity was attributed to the carbon of the ABS polymeric material, while titanium also existed at a slight concentration, confirming the previous claim that no obvious TiN agglomerations were formed. Moreover, EDS analysis did not show the existence of other elements, indicating the absence of impurities or moisture. 

Figure 14F shows a high-magnification (50,000×) SEM image of the side surface of the ABS/TIN 8 wt. % nanocomposite specimen. As can be observed, agglomerations of TiN nanoparticles and nanovoids were detected. EDS was carried out in the region where the agglomeration of the TiN nanoparticles appeared to be located close to a nanovoid (Figure 14G). The elemental analysis confirmed that the highest peak was attributed to Ti, while the carbon and nitrogen elements were validated. EDS also detected oxygen and chlorine at quite low intensities, which possibly originated from the TiN nanopowder, as shown by the EDS analysis (and it is in agreement with the TiN nanopowder composition provided by the manufacturer) (Figure 2B). However, it is also quite possible that the existence of a nanovoid induces the absorption of moisture, because the SEM image in Figure 13F shows some microporosity on the surface.

## 4. Discussion

**Figure 15:**
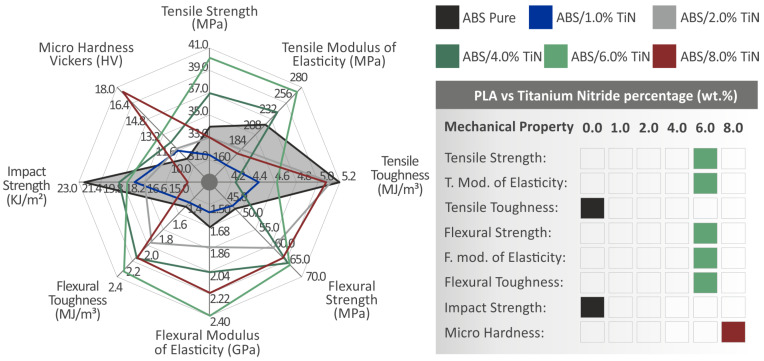
On the left side, the spider diagram depicts all the mechanical properties of the tested 3D-printed specimens. The grey area is representative of the pure ABS material, which was prepared and tested in the study, to be used as the benchmark material for the evaluation of the reinforcement performance. The table on the right presents the materials that exhibit the best mechanical response in each test.

Figure 15 summarizes the results of all the mechanical tests conducted in this work for the investigated nanocomposites and ABS pure polymer. The reference material for the evaluation of the reinforcement performance was pure ABS, which was also fabricated and tested within the context of the work, using the same material and conditions as the nanocomposites prepared herein. The enhancement mechanism of the addition of nanoparticles is generally due to and affected by the interaction between the matrix and the filler [71], the change in the matrix rheology [72], and the chemistry developed in the contact area between the nanoparticles and the matrix [73]. Compared to other additives [10,38,74] induced in the ABS matrix, the reinforcement in the mechanical properties by the addition of TiN nanoparticles was higher.

A nanocomposite with 6 wt. % TiN concentration demonstrates the best mechanical performance since most of the mechanical properties are reinforced. The greatest enhancement in the ABS/TIN 6 wt. % specimens are presented in the flexural properties, while sufficient improvements are also observed in the tensile strength and stiffness tests. These results were in good agreement with the tensile tests of the filaments produced. Impact strength and tensile toughness are the only properties that present an overall reduction in the mechanical response with the addition of TiN nanoparticles in the polymeric matrix. Moreover, it is important to note that most of the mechanical test results present a threshold of 6 wt. %, which means that the optimization of the mechanical properties follows a gradual trend as a function of the TiN concentration up to this point. However, the microhardness presents an advantageous continuous increase up to 8 wt. % filler loading, reaching a great percentage of 62.7%. The amount of TiN seems to have a direct impact on the enhancement of the developed nanocomposites, except for its superior hardness.

Excluding the case of microhardness, the decay of the performance of most of the mechanical properties is indicative of the ABS/TiN 8 wt. % specimen, especially in tensile and flexural tests. This decay was also observed in the tensile test of the produced filament. SEM images revealed that as the filler concentration increased in the ABS matrix, more and larger voids in both micro- and nano-scales were observed on the surface of the 3D-printed specimens. Notably, the size of the microvoids for the ABS/TiN 8 wt. % is much larger than that of other specimens with less filler content, while defects and micro-welds are also identified only on the side surface of the nanocomposite with the highest additive concentration. The agglomerations were located at 8 wt. %. Filler loading also negatively affects the mechanical performance of 3D-printed parts [75]. The inferior mechanical properties can also be attributed to reduced chain mobility at higher additive concentrations [38].

Therefore, the quality of 3D printing seems to be affected at this point; up to a concentration of 6 wt. %, the small size and distribution of voids do not have a negative impact on the mechanical behavior of the developed nanocomposites. It must be noted that the TGA measurements verify that the setting temperatures applied during the MEX process are not able to induce any degradation of the materials used, thus negatively affecting the 3D-printing process in general. The thermal stability of the polymeric matrix remained at the same level as that of the TiN nanoparticles. Moreover, comparing the data of the EDS analysis for the two nanocomposites with the highest concentrations, it was concluded that the agglomeration of the TiN nanoparticles started at a concentration of 8 wt. %. This indicates that the saturation threshold of the TiN nanoparticle distribution is at 6 wt. %, which seems to present a sufficient dispersion without the formation of agglomerations. Degradation of the mechanical behavior started above the saturation point of 6 wt. %, which also seems to strongly affect the deterioration of the morphological characteristics of the 3D-printed specimen. It was concluded that as the dispersion of the TiN nanoparticles in the ABS polymer matrix was maintained at sufficient levels, most of the mechanical properties were enhanced with an increase in the additive concentration, validating the beneficial feasibility of the MEX 3D-printing process for the development of ABS/TiN nanocomposites.

It should be noted that at a low TiN concentration of 1 wt. %, the mechanical properties were reduced and the reinforcement of the ASB polymer occurred at higher TiN loadings. This shows that the addition of the TiN nanoparticles in the ABS matrix does not show a univocal reinforcement effect on the polymer. The deterioration of the mechanical properties with the addition of nanoparticles is a possibility, as the literature reports [76]. With the addition of the nanoparticles, a decrease in the mechanical properties can occur at low concentrations and then the mechanical properties increase up to the point where the saturation of the additive in the matrix occurs [77]. This behavior can be attributed to weak interactions between the additive and the filler, which negatively affect the mechanical properties [78]. As the filler loading increases, the nanoparticles network is properly formed, and strong interactions between the matrix and the filler are established, leading to an increase in the mechanical properties [78].

Finally, it should be mentioned that the reinforcement of the ABS polymer with the proposed methodology by adding TiN nanoparticles in the matrix does not significantly compromise the cost of the final 3D parts production. The only cost in the process is the blending of raw materials, which can be evaluated as negligible, especially in industrial-scale applications. The remaining steps of the process for the MEX 3D-printing filament production were the same. Regarding the cost of the materials, there was an additional cost for TiN nanoparticles. Overall, the material cost for industrial-scale production is not that high, considering that the ABS raw material costs 0.35 €/kg for large orders (1000 kg) and the cost of the corresponding filament is approximately 25 €/kg. TiN nanoparticles cost approximately 0.9 €/gr for laboratory-scale applications. This cost can be significantly reduced for industrial-scale applications. ABS for laboratory-scale applications cost approximately 10 €/kg, which is 0.001 €/g. Optimum mechanical properties were achieved with a 6 wt. % loading nanocomposite. Thus, the cost per gram is 0.09 × 0.06 = 0.054 €/gr for the TiN particles and overall 0.001 €/g + 0.054 EUR/gr = 0.055 €/gr for laboratory-scale use. This increase in the cost of raw materials is high, but the effect on the overall cost of the filament production process cannot be considered significant.

## 5. Conclusions

This study investigates the capability of the TiN ceramic as an enhancement agent for the ABS polymer in MEX 3D printing. The development of ABS-based nanocomposites was demonstrated by introducing TiN nanoparticles at various concentrations, up to 8 wt. % in the polymeric matrix. The impact of nano-additive inclusions on the mechanical behavior of thermoplastic polymers was investigated thoroughly by conducting a range of mechanical tests on the developed 3D-printed specimens. The impact strength, microhardness, and tensile and flexural properties were measured under international standards, while tensile tests were also carried out on the filaments produced with the thermomechanical extrusion process.

The hypothesis of the study was proven. The results showed that the addition of TiN nanoparticles enhances the mechanical performance of the polymeric matrix. Most of the mechanical properties present remarkable improvements at an optimum concentration of 6 wt. % TiN in comparison with those of the pure ABS polymer. The highest increase compared to the pure ABS was observed in the flexural properties (flexural strength: 36.9%, flexural modulus of elasticity: 42.3%, and flexural toughness: 54%), whereas improved values were obtained for the tensile strength and stiffness of 18.1% and 22.2%, respectively. The microhardness of the nanocomposites presents a continuously improved trend as a function of the TiN content, reaching a high increase of 62.7%, affected directly by the high hardness of the additive material.

Thermogravimetric analysis showed that the thermal stability of the thermoplastic polymer was not affected by the inclusion of the TiN nanoparticles, remaining at the same levels across the ABS/TiN concentrations studied. This confirms the compatibility of the investigated materials with the methodology followed and the set temperatures and conditions applied in all the steps of the process. The maximum set temperature of the 3D-printing process is much lower than the starting temperature of weight loss at 345 °C. Raman spectroscopy measurements did not show changes in the bonds of the polymeric matrix or the chemical reaction of ABS with TiN nanoparticles.

On the other hand, the SEM images reveal that the morphological characteristics of the developed 3D-printed specimens are strongly affected by the inclusion of the nano-additive, creating voids on the micro- and nano-scales. The size and distribution of the voids in both scales increased with increasing the TiN concentration in the polymeric matrix. As a result, micro-porosity and defects were detected on the surface of the samples made with the nanocomposite with the highest TiN content, which is probably responsible for the decline in the mechanical performance above 6 wt. % TiN concentration in most mechanical tests.

High-magnification SEM images revealed nanoparticle agglomerations in the samples, which clearly had a negative impact on the morphology of the 3D-printed specimens. EDS analysis confirmed the presence of TiN agglomerations on the surface of the ABS/TiN 8 wt. % samples. In contrast, no nanoparticle agglomerations were formed on the surface of the ABS/TiN 6 wt. % samples, which strongly suggests that the saturation point of TiN nanoparticles dispersion in the ABS matrix is between 6–8 wt. %. This is reflected in the mechanical tests, explaining the performance optimization and threshold observed in most mechanical properties at the 6 wt. % filler loading. In conclusion, this report validates the feasibility of MEX 3D printing for the development of mechanically reinforced ABS/TiN nanocomposites. It is another successful paradigm that expands its applications and opens new routes for the powerful abilities of this AM technology. In future work, the exact saturation threshold of TiN nanoparticles in the ABS matrix can be identified, and additional mechanical tests can be performed to further analyze and optimize the enhancing effect of the TiN additive in MEX 3D printing.

## Figures and Tables

**Table 1:** Mechanical Characterization Tests.

Tensile	
Sample	Type V with a thickness of 3.2 mm
Strain rate	10 mm/min
Standard	ASTM D638–02a
Device	Imada MX2 (Northbrook, IL, USA)
Flexural	
Test Type	Three-point bending
Span length	52 mm
Strain rate	10 mm/min
Standard	ASTM D790
Device	Imada MX2 (Northbrook, IL, USA)
Impact	
Test Type	Charpy
Samples	Notched
Release height	367 mm
Standard	ASTM D6110
Device	Terco MT 220 (Kungens Kurva, Sweden)
Microhardness	
Method	Vickers
Applied load to the specimen during the indentation	200 gF
Indentations’ duration	10 s
Standard	ASTM E384–17
Device	300 Innova Test (Maastricht, The Netherlands)

**Table 2:** Summary of the mechanical test results.

	Pure ABS	ABS/TiN 1 wt. %	ABS/TiN 2 wt. %	ABS/TiN 4 wt. %	ABS/TiN 6 wt. %	ABS/TiN 8 wt. %
Tensile stregth (MPa)	34.0	31.5	33.0	36.9	40.1	33.0
Tensile modulus of elasticity (MPa)	223.2	167.2	185.7	214.9	272.8	179.4
Tensile toughness (MJ/m^3^)	5.2	4.5	5.1	4.2	4.6	5.1
Flexural stregth (MPa)	48.3	47.7	60.7	65.5	66.1	63.9
Flexural modulus of elasticity (GPa)	1.7	1.6	1.8	2.0	2.4	2.2
Flexural toughness (MJ/m^3^)	1.5	1.5	2.0	2.2	2.3	2.2
Impact strength (kJ/m^2^)	22.7	18.9	18.3	20.1	19.5	15.1
Microhardness (HV)	10.8	11.7	11.8	12.5	13.4	17.5

## Data Availability

The data presented in this study are available upon request from the corresponding author.
